# Polymeric Packaging Applications for Seafood Products: Packaging-Deterioration Relevance, Technology and Trends

**DOI:** 10.3390/polym14183706

**Published:** 2022-09-06

**Authors:** Yeyen Laorenza, Vanee Chonhenchob, Nattinee Bumbudsanpharoke, Weerachet Jittanit, Sudathip Sae-tan, Chitsiri Rachtanapun, Wasaporn Pretescille Chanput, Suvimol Charoensiddhi, Atcharawan Srisa, Khwanchat Promhuad, Phanwipa Wongphan, Nathdanai Harnkarnsujarit

**Affiliations:** 1 Department of Packaging and Materials Technology, Faculty of Agro-Industry, Kasetsart University, 50 Ngam Wong Wan Rd., Latyao, Chatuchak, Bangkok 10900, Thailand; 2 Department of Food Science and Technology, Faculty of Agro-Industry, Kasetsart University, 50 Ngam Wong Wan Rd., Latyao, Chatuchak, Bangkok 10900, Thailand

**Keywords:** food packaging, active packaging, indicator, advanced material, seafood

## Abstract

Seafood is a highly economical product worldwide. Primary modes of deterioration include autolysis, oxidation of protein and lipids, formation of biogenic amines and melanosis, and microbial deterioration. These post-harvest losses can be properly handled if the appropriate packaging technology has been applied. Therefore, it is necessary for packaging deterioration relevance to be clearly understood. This review demonstrates recent polymeric packaging technology for seafood products. Relationship between packaging and quality deterioration, including microbial growth and chemical and biochemical reactions, are discussed. Recent technology and trends in the development of seafood packaging are demonstrated by recent research articles and patents. Development of functional polymers for active packaging is the largest area for seafood applications. Intelligent packaging, modified atmosphere packaging, thermal insulator cartons, as well as the method of removing a fishy aroma have been widely developed and patented to solve the specific and comprehensive quality issues in seafood products. Many active antioxidant and antimicrobial compounds have been found and successfully incorporated with polymers to preserve the quality and monitor the fish freshness. A thermal insulator has also been developed for seafood packaging to preserve its freshness and avoid deterioration by microbial growth and enzymatic activity. Moreover, the enhanced biodegradable tray is also innovative as a single or bulk fish container for marketing and distribution. Accordingly, this review shows emerging polymeric packaging technology for seafood products and the relevance between packaging and seafood qualities.

## 1. Introduction

In 2021, world fishery market trade was forecast to increase by 12% in value and 3.7% in volume, with production projected to increase by 4 million tons from 2020 to 2022 [1]. Fishery product prices, especially shrimp, remained stable until July 2021 and then increased from August 2021 due to high freight costs of US$ 0.70–0.80 per kg for products exported from Asia to North America and Europe. Processed seafood is popular as ready-to-eat meals or snacks equipped with detailed serving instructions that normally involve reheating using a microwave oven [2]. Consumption of frozen packaged foods had predicted a CAGR of 5.98% from 2021 to 2028, while average year-on-year growth of 15.35% in 2020 was attributed to consumer panic buying behavior during the COVID-19 outbreak. Cold chain distribution requires high maintenance of both containers and packaging systems to maintain product quality. Seafood is a rich source of nutrients with specific aromas and tastes, while increasing global seafood production reflects consumer demand for packaged seafood products.

Packaging technology has been introduced to improve the quality with many beneficial solutions that offer interaction between food, packaging, and the environment. Finally, the shelf-life can be prolonged. Numerous types of research have investigated the efficacy of active and intelligent packaging, modified atmosphere packaging, as well as insulator packaging with various functions, forms, and materials/sources to preserve seafood quality. Active packaging focuses on inhibiting seafood deterioration, such as microbial growth and chemical oxidation. Intelligent packaging is designed to monitor the freshness of seafood products by detecting acidity, ammonia, or biogenic amine. Insulator packaging is for maintaining the temperatures in cold chain distribution. Active packaging is starting to be commercially developed. In the United States, active packaging contributed to 1% of the total packaging. This lower industrial scale of production relates to safety and legislative concerns [3].

Recent reviews relating to seafood technology include edible films and coating [4], spoilage and microbiota [5], and indicators for freshness [6]. However, an up-to-date review on polymeric and packaging technology for seafood remains lacking, especially after the COVID-19 outbreak. Recent packaging advances have focused on solving specific and comprehensive problems of seafood deterioration, such as mitigating the fishy smell; preventing and detecting biogenic amine, pH values, and ammonia; retarding melanosis formation and innovations in rigid packaging; as well as seafood product–package interaction. This review focuses on recent developments in polymeric-based seafood packaging with particular relevance between product and packaging.

## 2. Major Challenges for Seafood Packaging

### 2.1. Seafood Quality and Freshness

Seafood products (fish, crustaceans, bivalves, and cephalopods) are categorized as perishable, with high water content promoting rapid deterioration, especially by microorganisms. Improper handling during pre- and post-harvest accelerates the growth of indigenous microorganisms that trigger chemical and biochemical reactions that lead to deterioration. Water activity, the packaging system, storage temperature, and hygiene are the main external factors that influence seafood quality. Appropriate packaging technology is required to preserve the quality of seafood products during storage and bulk or retail distribution. Recent designs of active packaging, intelligent packaging, and modified atmosphere packaging (MAP), as well as thermal insulated packaging, maximize shelf-life while inhibiting factors of deterioration.

Packaging mainly functions to protect the product from undesirable environmental conditions such as heat, oxygen, light, or contamination (biological, chemical, and physical) with the required water and gas barrier, mechanical, optical, thermal, and physical properties. Seafood packaging is designed to preserve product quality. Active packaging inhibits microbial growth in seafood products. High barrier packaging/vacuum or high CO_2_ packaging is used to prevent lipid and protein oxidation. Intelligent packaging is designed to monitor seafood freshness according to biogenic amines, pH changes, and ammonia production. Insulator packaging is designed to maintain low temperatures during seafood distribution.

### 2.2. Fishery Smell in Seafoods

The fishy aroma of seafood is the major factor impacting consumer acceptance. The aroma of seafood depends on the habitat, origin, type of nutrition content, breed, and processing method [7]. Freshwater fish have a mud-like aroma due to the presence of geosmin, while oct-1-en-3-ol, hexanal, and heptanal are chemical compounds responsible for the fishy odor [8]. Volatile organic compounds responsible for the seafood-like aroma include alcohols, aldehydes, ketones, esters, and sulfur, which are found in *Ruditapes phillipinarum* (Manila clam) [7]. The fishy aroma of seafood products is also related to polyunsaturated fatty acids (PUFAs) as the dominant fatty acid content in seafood. PUFAs can be converted into derivatives of unsaturated aldehydes including 1,6-nonadienal, 2,4-decadienal, and 2,4,7-decatrienal with a low odor threshold concentration [9]. Trimethylamine is the major source of the fishy smell in seawater fish, which gets stronger during storage due to post-harvest metabolism.

Managing the fishy-like aroma is challenging during transportation and storage. Oxidation of proteins and lipids in fish results in undesirable odors that need to be contained by high gas barrier packaging, or MAP. PVOH and nylon block the diffusion of fishy smells from inside to outside the packaging, thereby preventing odor pollution and product oxidation. Kimbuathong et al. (2020) [10] reported that MAP with a high CO_2_ concentration reduced shrimp TMA production. The fishy-like aroma originating from some volatile compounds may also react with the packaging, limiting the reuse and recycling of Styrofoam box-based thermal insulators. Ishida (2020) [11] reported that trimethylamine sticks to Styrofoam after use and can be dissolved in vegetable oil to improve its recycling potential.

The application of bio-based packaging for seafood products needs to be improved to contain the fishy aroma of seafood because the barrier properties are relatively lower than conventional packaging. Ortega-Toro et al. (2016) [12] improved the barrier properties of thermoplastic starch by blending with poly(ε-caprolactone), while Bang and Kim (2012) [13] improved the barrier properties of poly(lactic acid) (PLA) by insertion into an inorganic silica network as a hybrid coating material via the sol-gel method. Dong et al. 2022 [14] synthesized poly(butylene glycolate-*co*-furan dicarboxylate) with excellent barrier properties 68.6 times higher than poly(butylene adipate-*co*-terephthalate) (PBAT), while Katekhong et al. (2022) [15] improved the gas barrier properties of thermoplastic starch/PBAT based film by adding nitrite as a plasticizer and active agent. The permeability of thermoplastic starch/PBAT blends depends on the hydrophobicity of starch, and octenyl-succinated starch reduced the barrier properties more than acetylated starch [16].

Incorporation of essential oils with highly desirable smells such as lime, ginger, garlic, or oregano into the packaging also reduces the fishy aroma of seafood. Essential oils are aromatic organic compounds extracted from plants that exist in various forms, including aldehydes, alcohols, ethers, ketones, acids, amines, and other volatiles. Seafood products contain high amounts of fat and moisture that increase the release of phenolic compounds from essential oils such as aliphatic hydrocarbons (8–10 carbon atoms) in citrus oil, aliphatic molecules (6 carbon atoms) in leafy-green scented floral oils, or octanal aldehyde in orange oil, which are responsible for odor migration from packaging to seafood [17]. The patent for removing the fishy smell of salted mackerel using peanut sprouts was listed by Yun Hee and Kgeong Su (2018) [18]. They claimed that salted mackerel treated with peanut sprout extract had a lower fishy smell and trimethylamine content (0.21 Mg%) than untreated salted mackerel (1.91 Mg%). Another patent listed by Yoo (2014) [19] involved the use of garlic pickle and tangerine peel to remove the fishy smell of mackerel and hairtail stew. The fishy smell is an issue regarding consumer acceptance and air pollution. Nevertheless, it can be controlled via packaging with a desirable smell and prevented by high barrier packaging.

## 3. Packaging Related Deteriorations of Seafoods

### 3.1. Microbial Deterioration

Microorganisms are mainly responsible for seafood deterioration and emanate from the internal body or the environment. The most dominant spoilage bacteria found in fish include *Pseudomonas* and *Achromobacter* spp. [20]. The microbial flora of seafood is also affected by the habitat. Microbial genera such as *Vibrio*, *Enterobacter*, *Serratia*, and *Aeromonas* were dominant in the freshwater fish (*Pangasius pangasius*), while *Vibrio fluvialis*, *Proteus mirabalis*, *Proteus vulgaris*, *Shewanella putrefaciens*, and *Ochrabactrum anthropic* were found in seawater fishes (*Lutjanus sanguineus* and *Lates calcalifer*) [21]. Specific spoilage bacteria produce different compounds depending on the targeted nutrition. Aerobic spoilage produces ammonia, acetic or propionic acids, while anaerobic microorganisms and yeasts produce ketones, esters, aldehydes, ammonia, and acids that contribute to the fish aroma. Hydrogen sulfide (H_2_S) producing bacteria such as *Shewanella putrafaciens*, *Vibrionaceae*, and lactic acid bacteria also contribute to fish deterioration. *Photobacterium phosphoreum* together with H_2_S spoilage bacteria trigger the production of trimethylamine during post-harvest metabolism. Production of hypoxanthine from the fish body during storage is triggered by *Enterobacteriaceae*, *Pseudomonas*, *Shewanella putrafaciens*, and *Photobacterium phosphoreum.*

**Figure 1:**
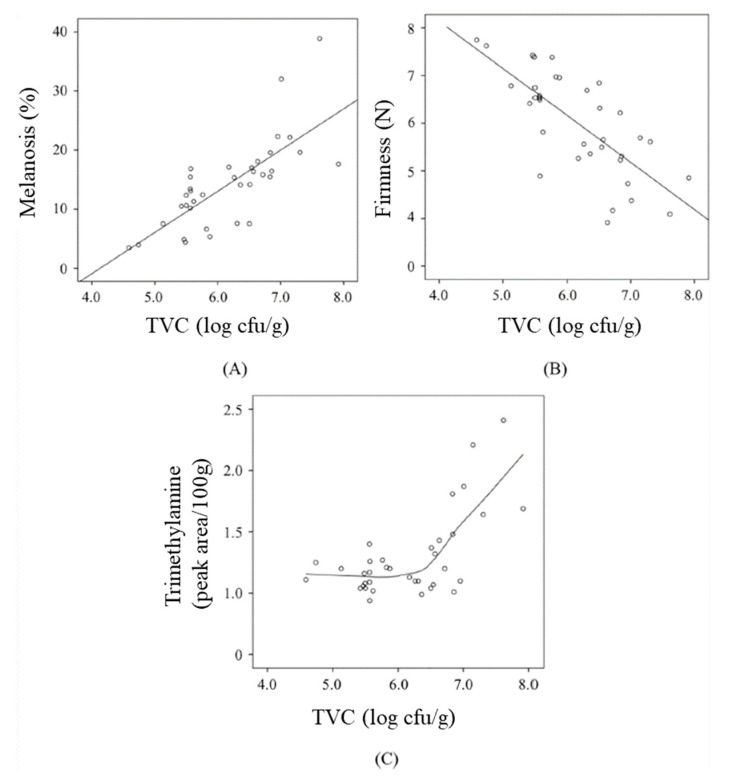
Interrelation of total viable count on changes in (**A**) melanosis, (**B**) firmness, and (**C**) trimethylamine content of shrimp under a modified atmosphere during 12 h of storage at 4 °C (Reproduced with permission from Kimbuathong et al., 2019 [10]).

**Figure 2:**
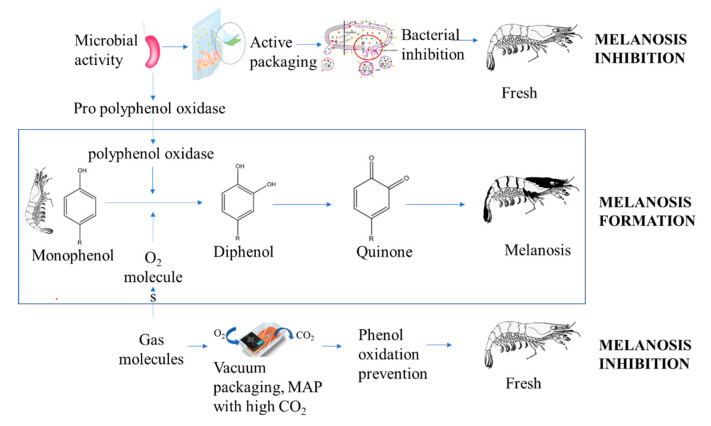
Melanosis formation through polyphenol oxidation and its prevention via microbial inhibition and O_2_ removal using packaging technology.

The effect of microbial growth on changes in other fish quality parameters was reported by Kimbuathong et al. (2019) [10]. Microbial growth was linearly correlated with melanosis and trimethylamine production of Pacific white shrimp stored under MAP (Figure 1). This finding indicated that microbial growth strongly stimulated melanosis formation in shrimp following the mechanism shown in Figure 2, while the peptidoglycan binding protein (Gram positive bacteria) and β-1,3-glucan binding protein (Gram negative bacteria) contributed to pro-polyphenol oxidase activation that then induced melanosis formation [22]. Trimethylamine dramatically increased after the total viable count (TVC) reached 6.8 log cfu/g. These results suggested that reduction of trimethylamine and melanosis was dependent on the microbial inhibition ability of CO_2_. Increasing TVC also inversely reduced the firmness. The microbials consumed nutrients such as lipids or proteins, causing proteolytic denaturation and resulting in a loss of firmness.

Several studies have investigated microbial inhibition in seafood products using active packaging. Mohebi and Shahbazi (2017) [23] found that chitosan film containing *Ziziphora clinopodioides* essential oil and pomegranate peel extract effectively retarded TVC and growth of *Pseudomonas* spp., *Pseudomonas flourescens*, *Shewanella putrefaciens*, Enterobacteriaceae, lactic acid bacteria, and *Listeria monocytogenes* in shrimp compared to the control and gelatin film, while incorporation of combined nanoparticles (ZnO, SiO, and CuO) into gelatin and polyvinyl alcohol film had antimicrobial activity equal to the positive control against *S. aureus*, *L. monocytogenes*, *E. coli*, *P. fluorescens*, *V. parahaemolyticus*, and *A. caviae* [24]. Microbial activity in seafood products can be controlled via active packaging containing antimicrobial agents that delay post-harvest metabolism and extend the shelf-life.

### 3.2. Chemical Deterioration

Deterioration in seafood is mainly caused by lipid oxidation in high-fat content pelagic fish (mackerel, sardinella, and herring). Oxidation occurs as a result of the reaction between oxygen molecules and the double bond of the unsaturated fatty acid chain. Fish contain high amounts of mono or polyunsaturated fatty acids such as eicosapentaenoic acid (EPA) and docosahexaenoic acid (DHA) as major sources of nutrition benefits that are highly susceptible to oxidation. Packaging containing antioxidant agents, such as carvacrol essential oil [25], gallic acid, and clove essential oil [26], loaded polymer reduced lipid oxidation of salmon fillets. Li et al. (2021) [27] reported that MAP combined with ε-polylysine, chitosan, and sodium alginate coatings delayed myofibril oxidation by inhibiting carbonyl groups in pufferfish.

Lipid oxidation in fish occurs enzymatically or non-enzymatically. Enzymatic lipid oxidation results from lipolysis by lipases, while non-enzymatic oxidation normally arises in hematin compounds such as hemoglobin, myoglobin, and cytochrome [28]. Myoglobin is present in red meat such as tuna as an oxygen-binding protein similar to hemoglobin. High myoglobin content contributes to the reddish-brown color of the flesh. Undesirable discoloration in red meat is related to lipid oxidation associated with the binding between heme and myofibrillar protein and results in greater discoloration [29]. The red color in tuna can be preserved by using carbon monoxide or nitric oxide that react with myoglobin (MB), resulting in MB-Fe^+11^-CO and MB-Fe^+11^-NO bonding, respectively [30]. Therefore, chemical deterioration depends on the chemical structure of the fish meat, while preservation is accomplished using an antioxidant agent or high CO_2_ atmosphere.

### 3.3. Biochemical Deterioration

Post-mortem biochemical changes in fish result from autolysis by the action of proteolytic enzymes. Ghaly et al. (2010) [28] explained that proteolytic enzymes are mostly present in the muscle and viscera of fish in the early rigor stages and contribute to post-mortem degradation. Proteolytic enzymes break down protein, resulting in free amino acids and peptides during autolysis that lead to fish degradation, with production of the biogenic amines: histamine, tyramine, tryptamine, putrescine, and cadaverine [31], followed by acidity, increased pH, and accumulation of basic nitrogen compounds such as trimethylamine (TMA) and total volatile-based nitrogen (TVB-N). The degradation of adenosine triphosphate (ATP) is also responsible for the biochemical post-mortem degradation of seafood products to form adenosine diphosphate (ADP), adenosine monophosphate (AMP), inosine monophosphate (IMP), inosine, and hypoxanthine, determined as K value [32]. A higher K value indicates a lower freshness level.

Kimbuathong et al. (2019) [10] reported that MAP with a high concentration of CO_2_ (above 60%) retained a low TMA value. A combination of MAP and ε-polylysine, chitosan and sodium alginate coatings maintained Ca^2+^-ATPase activity by retaining the protein structure (α and β-sheet) of pufferfish [27], while TVB-N and TMA of salted dried Atlantic mackerel were reduced by vacuum packaging combined with an iron-based oxygen absorber [33]. Biochemical deterioration mostly relates to the amine content in fish meat, and this can be delayed by MAP or active packaging.

### 3.4. Melanosis

Melanosis occurs when a cell cannot regulate pigment production and appears as a black spot. This is a serious issue in crustacean products considering their high commercial value. Melanosis is harmless to human health but significantly decreases sensory market value and acceptance by the consumer. Goncalves and Oliveira (2016) [22] categorized five stages of melanosis formation: (1) Gram positive and negative bacteria and fungi trigger the alignment of serine proteinase, (2) pro polyphenoloxidase then activates to become polyphenoloxidase (PPO), (3) PPO acts as a catalyst to convert phenol into colorless quinone, (4) oxidization of quinone results in melanin, and (5) melanin is responsible for melanosis formation in the crustacean carapace. Melanosis formation is accelerated by microorganisms, oxygen exposure, and high temperatures. Melanosis in crustaceans can be measured by PPO extraction and identification using SDS polyacrylamide gel electrophoresis (SDS-PAGE) [34], color measurement, particularly whiteness [35] or L value [36], image analysis [10,37], or sensory assessment [38,39].

Packaging systems integrated with active compounds are now widely developed to preserve crustaceans, particularly shrimp, by inhibiting melanosis formation on the carapace. MAP with a high CO_2_ concentration to prevent melanosis in shrimp [10,35], while Laorenza and Harnkarnsujarit (2021) [37] developed a PBAT/PLA active film containing carvacrol, citral, and α-terpineol essential oils to prevent melanosis. Alparslan et al. (2016) [36] also reported that edible coatings prepared from gelatin containing orange leaf oil retained a high L* value, indicating that melanosis was prevented. Application of packaging technology prevented melanosis in shrimp by minimizing oxidation and growth of melanosis-stimulating microbes.

## 4. Polymeric Packaging for Seafoods

### 4.1. Conventional Polymer-Based Packaging

Conventional polymer-based packaging, including polypropylene (PP), polyethylene (PE), polyethylene terephthalate (PET), polystyrene (PS), polyvinyl alcohol (PVOH), and polyamide (PA), still dominates the packaging industry with excellent mechanical, barrier, and thermal properties compared to plastic from other sources. Seafood is usually produced as frozen products, with a temperature during freezing of less than −40 °C and a frozen storage temperature −18 °C. Frozen seafood packaging must withstand low temperatures without cracking. Most frozen products become hard with sharp edges under freezing conditions due to their transition into an amorphous glassy state, causing impingement through flexible films and loss of package integrity. Glass transition temperature (Tg) plays a key role in the choice of packaging material properties. Polymers with higher Tg than freezing and storage temperatures are unsuitable for use with frozen products. Polyethylene has low glass transition temperature at up to −100 °C compared to other petroleum-based polymers, suggesting that it would be a suitable packaging material for products stored at extremely low temperatures [40]. Petroleum-based packaging for seafood products is usually in a flexible (pouch, bag, or pocket) or rigid (tray or box) form. Flexible packaging is made from PP, PE, or nylon due to the high elongation at break, while rigid packaging is normally made from PET or PS due to poor elongation at break.

The development of conventional polymer-based packaging for seafood products includes functional polymeric packaging and MAP that delays microbial growth. Dong et al. (2018) [41] found that the incorporation of rosemary and cinnamon essential oils into polyethylene reduced the degradation temperature and barrier properties of polypropylene film because the hydroxyl groups reacted with oxygen or water molecules and retarded shrimp deterioration. Nylon has excellent barrier properties and is mostly used as vacuum packaging in processed seafood products. Vacuum packaging with very low oxygen permeability maintained seafood quality by removing undesirable gases to minimize post-harvest metabolism [42], while incorporation of anthocyanin originating from plants with polyvinyl alcohol could monitor the freshness of shrimp [43].

Rigid and semi-rigid packaging such as PET-based trays play an important role in fishery product marketing, particularly in retail or convenience store fresh produce displays. Reduction of water-holding capacity in muscle structures causes high-drip loss of seafood products. This adversely affects appearance and consumer satisfaction. Liquid absorption pads are commonly used in the meat industry but are not popular in seafood packaging. The water absorbance pad is placed in direct contact with the fresh product and the water is still in contact with the product. Many patents of water-absorbent discs based on PLA modified material provided indirect contact water absorption between the fresh product and the absorption pad [44]. They designed an outer dish body and a depository dish body in an overlapped mode containing a water accumulation cavity between them. The water absorption sponge is placed inside the water accumulation cavity. The water absorption sponge has a lower size than the height of the accumulation cavity so that the system allows for the absorption of the water in irreversible contact with the product. Rigid packaging is also used to reheat food, and PET, PP, PE, and PS are suitable for microwave heating. A tray can also be used as a seafood MAP to maintain quality and mitigate oxidation and microbial growth.

Thermal insulation is also important for seafood packaging. Seafood is highly prone to microbial and enzymatic degradation, and temperature control is important to maintain product quality. Polystyrene-based rigid packaging such as a tray or box is usually used as a thermal insulator in seafood product delivery to maintain low temperatures. Several types of box insulation packaging have been commercially produced and registered as patents such as insulator bags made from glass wool and non-woven fabric [45], rectangular parallelepiped-based insulator boxes with sodium polyacrylate [46], intermediate foam fish boxes from olefin [47], Styrofoam boxes with intermediate lids [48], and insulator boxes with inflatable bags and wood pulp-based absorbance as wave-shaped convex strips [49]. Recently, conventional packaging still dominates in the packaging industry due to its excellent properties, processability, and lower price compared to bio-degradable polymers. However, end-use disposal of conventional polymer-based packaging is a serious problem; they are non-biodegradable due to their complex chemical structure of stable carbon–carbon linkages and are not broken down by microorganisms. Blending with biodegradable materials with high compatibility will improve the biodegradability without interfering with the properties.

### 4.2. Bio-Based Packaging

The majority of bio-degradable packaging originates from natural biopolymers and synthetic degradable polymers such as polysaccharide-based materials, which are environmentally friendly in terms of disposal and end life. Starch is one of the primary materials used in bio-degradable packaging, which is a renewable source derived from plants [50]. Starch is dominated by a hydroxyl group, resulting in a rigid network [51] and can be molded according to desirable shapes [52]. Thus, it is suitable for rigid packaging such as a tray, which is now dominated by a conventional polymer. The tray is commonly used to protect food from damage caused by undesirable environmental conditions such as shock, vibration, pressure, and deformation.

Polysaccharide-based trays or foam were developed to replace Styrofoam as an environmentally friendly packaging. A patent regarding a starch-based foam tray with at least two slots in the tray body combined with two fasteners, on which the tray body can be stacked and mutually form-embedded through the tray’s grooves and fasteners. This tray contains plurality holes, can accommodate loads up to 80 kg [53], and can be used as a fish bulk container. A patent was also registered for biodegradable resin foam sheets prepared from cellulose acetate as the main component with a starch and talc or eggshell powder-based modifier. This had high water-resistant properties as cup trays [54] for high water product packaging. Xiqing et al. (2021) [55] patented a biodegradable tray composited from starch, bamboo pulp, succinic acid, fiber, paper pulp fiber powder, calcium alginate fiber, milk protein fiber, polyethylene resin, and a toughening agent (polyvinyl alcohol or acrylate rubber), which automatically degrades after use.

Antimicrobial trays made of high-amylose corn starch and cinnamyl aldehyde were patented by Zhaohui et al. (2016) [56]. They developed the trays by ultrasonic waves without added surfactant to prolong product shelf-life due to the slow release of cinnamyl aldehyde. Mclaughlin and Yapp (2022) [57] patented a food packaging tray equipped with a sealed compartment using a gasket and cold-seal adhesive with no hot-sealing process. This packaging is to carry the reheated food from the store to keep the food safe and hygienic. They created the easy reseal system by reactivating the cold seal adhesive between the adhesive surface and another surface contacted together. Therefore, the tray plays an important role in seafood packaging, particularly in the selling and distribution, to keep products safe and hygienic for the consumer. Recently, starch-based rigid packaging has been widely developed and commercialized for tableware, packaging, cushioning, and insulation. The application of starch in this field still has limitations due to low water resistance since seafood products have high water content. Future research can be focused on improving the water resistance of starch by chemical modification, lamination, or blending with other materials that have great water resistance.

## 5. Innovative Polymeric Packaging Enhancing and Monitoring Seafood Quality and Freshness

### 5.1. Active Polymer Technology for Seafoods

Active packaging was described by Labuza in 1987 [58] as packaging, packaging materials, or packaging solutions that can interact with food and have a specific function beyond protecting food from the outside environment. The definition of active packaging was then expanded as technology advanced to face new problems. Nowadays, active packaging is defined as a packaging material that releases or treats substances from or into food or the environment. Active packaging methods can be divided into oxygen scavengers [59], carbon dioxide generating systems, ethylene scavengers, flavor and odor absorbers, antioxidants, and antimicrobials [60]. The technology of inserting the active compound into the packaging can be divided by coating [61], incorporating [41,62], surface modification [63], and adding directly into the packaging (i.e., essential oil into MAP) [64], which are able to modify and improve product quality, achieving desirable purposes [65]. The active agents are mostly applied in a polymer and effectively preserve the seafood quality (Table 1), including essential oils [23,37,66], plant phenolic compounds [67], or nano minerals [24,68].

**Figure 3:**
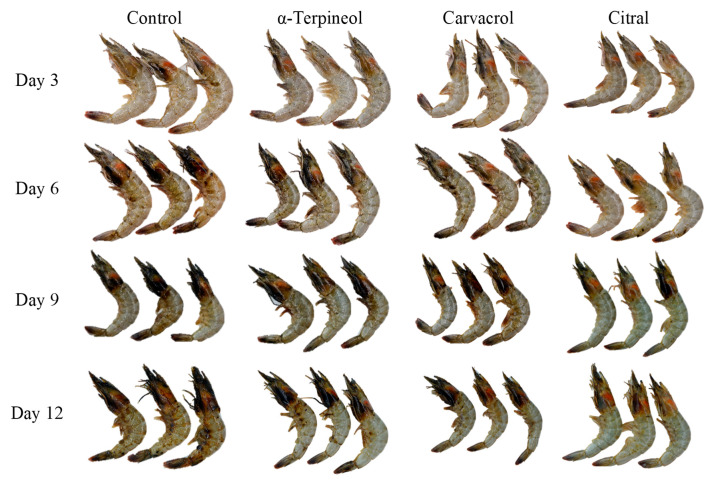
Shrimp packaged in PBAT/PLA film containing 6% α-terpineol, carvacrol, and citral during 12 days of storage at 4 °C.

The incorporation of essential oils in the polymer takes advantage of the volatility of antimicrobial agents that migrate from the packaging to the food inside. Laorenza and Harnkarnsujarit (2021) [37] found that incorporation of carvacrol, citral, and α-terpineol essential oils released from the PBAT/PLA matrix into the headspace. The release behavior of these essential oils was related to shrimp quality with lower microbial growth, lipid oxidation/thiobarbituric acid reactive substance (TBARS), and melanosis formation, while films containing essential oils delayed glycogen metabolism revealed in FTIR absorbance at wavenumbers 2800 to 3040 cm^−1^, which also agreed well with the protein degradation and head loss retardation. Shrimp packaged in films containing carvacrol, citral, and α-terpineol essential oils are shown in Figure 3.

*Ziziphora clinopodioides* essential oil and pomegranate peel extract in chitosan and gelatin film showed a synergistic effect, and were effective in inhibiting microbial growth in shrimp [23]. Essential oils were also applied in bilayer packaging to control the release direction of volatile compounds. Arancibia et al. (2014) [81] developed bilayer agar and alginate film containing cinnamon essential oil for chilled shrimp packaging. They found that cinnamon essential oil in agar was effective in inhibiting microbial growth due to essential oil interaction differences between agar and alginate. Dong et al. (2018) [41] also developed bilayer active packaging from low density polyethylene (LDPE) containing rosemary and cinnamon essential oils as an inside layer. They found that films containing combined rosemary and cinnamon essential oils were more effective in reducing TVB-N, TBARS, and microbial count. Essential oils containing strong antimicrobial and antioxidant activity were released from the packaging and brought into contact with the food to preserve quality.

**Figure 4:**
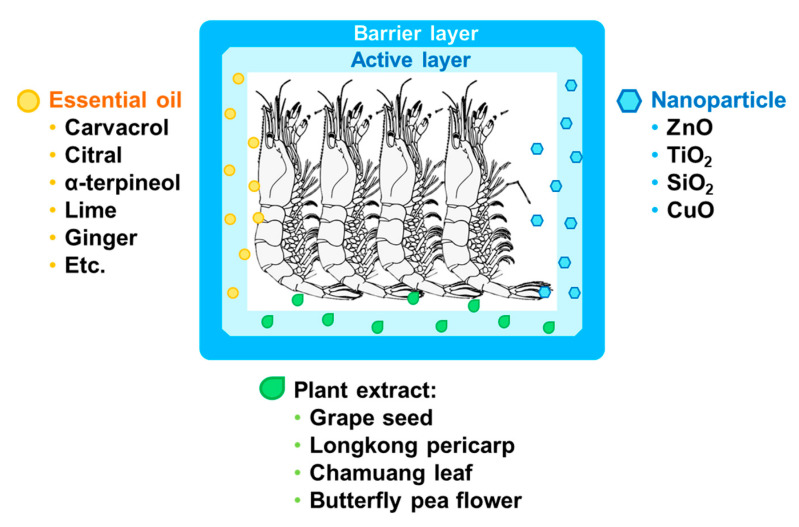
Active packaging with various active agents (essential oils, plant extracts, and nanoparticles) and its application for seafood products.

Plant extracts with antioxidant properties incorporated into polymers as seafood packaging were also reported (Figure 4). Lopes et al. (2021) [67] loaded potato peel extract onto starch film. The 2,2′-azino-bis-3-ethylbenzthiazoline-6-sulphonic acid (ABTS) inhibition of the film was measured at 56–85% within 7 days and increased the golden color of smoked sea bream fillet. Grapefruit seed extract incorporated with sodium alginate retarded the microbiological limit of shrimp for up to 4 days longer than the control, with lower TVB-N, pH values, and off-flavors due to dispersion of nanoparticles from the packaging to the shrimp [82]. Nagarajan et al. (2021) [83] developed an active coating prepared from gelatin and chitosan incorporated by longkong pericarp extract. They found that the active coating effectively inhibited melanosis and polyphenol oxidase activity of black tiger shrimp, while lipid (TBARS, peroxide value, and anisidine value) and protein oxidation (loss of sulfhydryl group) were inhibited. These results indicate that the incorporation of plant extracts rich in antioxidants into polymers has the potential to preserve the quality of shrimp. However, further investigation is required regarding the stability of plant extracts, particularly thermal and oxidative degradation.

Nanoparticles were also reported as a potent antimicrobial agent incorporated with polymers (Figure 4). With their small molecule size, large surface area, and reactivity via the hydroxyl group, nanoparticles are easy to insert into a polymer [84]. Silicon dioxide (SiO_2_) [85], zinc oxide (ZnO) [86], and titanium dioxide (TiO_2_) [87] are common nanoparticles used in food applications. Al-Tayyar et al. (2020) [84] reviewed the active functions of nanoparticles. They found that the semiconductive properties of nanoparticles were able to generate reactive oxygen species (ROS) and that Zn^2+^ antimicrobial ions were provided by nanoparticles in polar media. The nanoparticles interacted with carboxyl and amine groups on the bacterial membrane surface [88]. Another possibility was oxidative damage of the bacterial cell surface membrane by hydrogen peroxide by nanoparticles. Shao et al. (2021) [24] prepared active films from gelatin and polyvinyl alcohol containing SiO, ZnO, TiO, and copper oxide (CuO). They found that films with these nanoparticles significantly reduced the total viable *Shewanella putrefaciens*, Enterobacteriaceae, and *Pseudomonas* spp. counted in shrimp and *L. monocytogenes*, *S. aureus*, and *E. coli* inoculated from shrimp. Moreover, polyvinyl alcohol and gelatin film containing ZnO and TiO_2_ extended the shelf-life of shrimp by up to 6 days longer than the control [68].

Several patents have been registered regarding active packaging, including beta-cyclodextrin and essential oil inclusion compounded in polyvinyl alcohol [89]. They found no mold in the film after 18 days of incubation under 90% RH at 28 °C. A patent on multilayer active packaging was registered by Roberto et al. (2014) [90]. They developed a multilayer film consisting of paper (outer layer), polyethylene, or biodegradable plastic materials, an optional third metallic layer with an adhesive layer in the interposition, and an inner layer containing *Rosmarinus officinalis*, *Citrus limon*, or *Vitis vinivera* in contact with the packaged food. Results showed that the biogenic amine of fish samples was inhibited by up to 45%, 36%, and 39% after 2, 4, and 7 days of incubation, respectively. Obaiah et al. (2012) [91] developed another patent in active packaging as a dual function O_2_ absorbing and CO_2_ emission pouch for fishery products. Sodium bicarbonate, citric acid, and iron powder effectively absorbed O_2_ with less than 1% concentration remaining within 24 h, while CO_2_ remained high (80%) within 48 h. They explained that O_2_ absorption was based on the principle of iron oxidation, whereas CO_2_ emission came from the reaction between bicarbonate compounds and acids from citric acid releasing carbon dioxide along with the formation of other compounds. Furthermore, a patent for active amine scavenging for fish packaging was registered by Karlheinz and Francesca (2003) [92], consisting of at least one layer of ethylene with an unsaturated carboxylic acid group neutralized by a metal ion capable of adsorbing an undesirous amine from the headspace of the package. Active packaging is designed to have an active compound/system with the purpose of preventing deterioration factors and extending the shelf-life of food products. Active packaging has started to be commercialized; however, the production volume is still low due to safety and regulation. The low stability of active agents also becomes a major factor in causing the short shelf-life of active packaging. Future research focuses on protecting the active agent from damage such as oxidative and undesirable release amounts by microencapsulation as control release, lamination, or multilayer consisting of the high barrier layer.

### 5.2. Polymeric Sensors for Seafood

Intelligent packaging is defined as a system equipped with tools that are sensitive to environmental changes and continue to inform the users about the changes [93], while also providing information related to the function and properties of the packaged foods [94]. Intelligent packaging facilitates decision-making, warning of potential risks, food safety, and freshness [95]. Intelligent packaging can be divided into time-temperature indicators or gas indicators, biological sensors, humidity indicators, barcoding techniques, and radio frequency identification systems [96]. Food deteriorates during storage due to microbial activity, producing metabolites such as volatile amines and organic acids, which react and are then detected by the sensor-equipped intelligent packaging system that makes significant visible changes as a signal to assess the freshness level of the product [97]. Bioactive compounds extracted from plants have been widely investigated as freshness indicators in intelligent packaging. Bhargava et al. (2020) [97] compiled freshness indicators such as anthocyanin, curcumin, betalains, chlorophyll, carotenoids, tannins, quercetins, and brazilin, while chemical-based sensors incorporated with polymers were developed, including alizarin [98], pelargonidin [99], cyanidin-3-glucoside [100], and rhodamine B [101].

**Figure 5:**
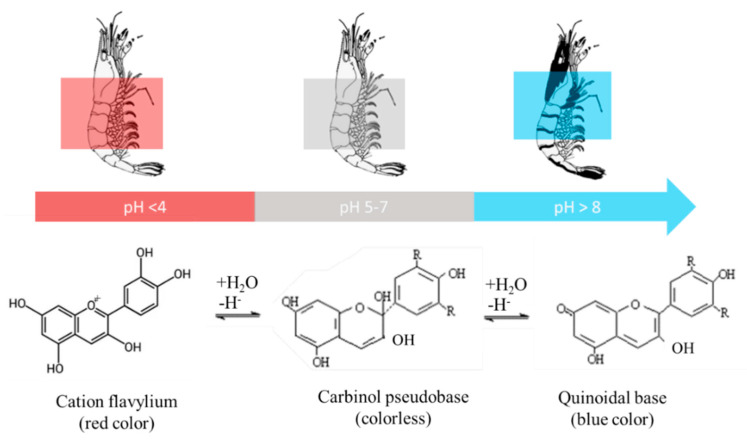
Mechanism of color change in pH−sensitive anthocyanins to monitor shrimp freshness (adapted from Zhao et al. 2022 [106] and You et al. 2000 [102]).

Biological/bioactive sensors are the most common intelligent packaging applied to seafood products, with metabolism outcomes such as ammonia, volatile nitrogenous compounds, and acidity (pH value) (Table 2 and Figure 5). You et al. (2022) [102] explained that anthocyanin loses the cations on the original oxygen atoms in C-ring and decreased the original salt ion concentration, resulting in a yellow color, and gradually turned blue in response to an increase in pH. Curcumin is susceptible to humidity changes and is suitable for use as a humidity indicator. Higher humidity promoted contact between ammonia and water molecules, resulting in ammonium cations and hydroxide, leading to a more alkaline environment and accelerating red color formation [103]. Metal-based colorimetric indicators such as gold, silver, or copper nanoparticles were also reported to be able to detect meat spoilage. Metal colorimetric indicators are suitable as volatile sulfuric compound detectors due to the high affinity between bonded metallic cations and sulfide anions. However, metal-based indicators, particularly Cu, were reported to have high stability in pH changes or no changes were found in variant pH values [104]. They also found that copper nanoparticles changed from dark yellow to red and finally black in response to an increase in hydrogen sulfide concentration in the fish. Fish spoilage results from the reaction of the volatile sulfur gas hydrogen sulfide with metals.

Common derivatives of anthocyanin include delphinidin, peonidin, pelargonidin, cyanidin, petunidin, and malvidin [99]. Pelargonidin was applied in intelligent packaging of tilapia fillets by Liu et al. (2021) [99]. They found that the orange-red color originating from the flavylium cation was reduced due to the formation of quinone, resulting in carbinol pseudobase formation. Absorbance increased with increasing pH from 7 to 10 as a result of the redshift phenomenon that commonly occurs in anthocyanin. Anthocyanin as cyanidin-3-glucoside was also applied in intelligent packaging for tilapia fillets. The red color at pH < 4 originated from the flavonoid cation and then turned colorless in the pH range of 5–6 due to the conversion of the flavonoid cation to carbinol pseudobase and chalcone, while cyanidin shifted to quinonoidal anhydrobase with a purple/blue color at pH 6–8 [100].

Dye-based colorimetric indicators have been used as potential sensors. Alizarin contains a phenolic hydroxyl group that can easily deproteinize in neutral pH, resulting in color changes from yellow to red, while the second deproteination of the phenolic hydroxyl group occurs in alkaline pH (9–11), resulting in a purplish red color [98]. Liu et al. (2022) [101] found that the AIE-stimuli-responsive polymer tetraphenylethylene/polymethacrylic acid (TPE/PMMA) with rhodamine B acted as an acidity-dependent indicator that changed polymer conformation by ionization of the carboxylic acid group, thereby exhibiting a change in fluorescence intensity. This indicator was more sensitive in response to amines (trimethylamine and dimethylamine) than ammonia in salmon. Intelligent packaging based on poly (3,4-ethylenedioxythiophene) and polystyrene sulfonate as flexible ammonia gas sensors for fish meat by coating them onto metal electrodes was patented by Yabo et al. (2022) [105]. The response value increased with increasing ammonia up to 94%, with humidity reaching 85%. Intelligent packaging now monitors food consumption safety using sensors that can detect various deterioration factors. The natural-based biosensor has a high potential as a dual-function biosensor as well as an antimicrobial agent to monitor the freshness and preserve the quality of seafood products.

## 6. Other Combined Technologies with Polymeric Packaging to Preserve Seafood Quality and Freshness

### 6.1. Modified Atmosphere Packaging for Seafood

**Figure 6:**
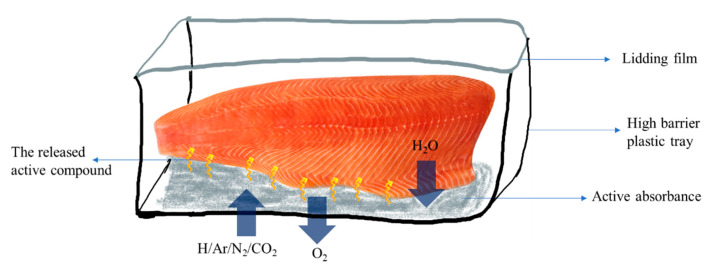
Application of various gases in modified atmosphere packaging with active absorbance in salmon product.

Modified atmosphere packaging (MAP) controls the gaseous atmosphere surrounding the food inside its packaging using specific polymeric packaging materials with appropriate levels and gas barriers to maintain gas transfer from and to the environment for food preservation (Figure 6) [117]. MAP is a non-thermal technology with multiple advantages for extending seafood shelf-life [35]. Lipid oxidation, protein degradation, microbial growth, and melanosis can be prevented by providing poor O_2_, rich CO_2_ [118], and argon (Ar) [119]. Gas concentration inside the MAP changes during storage due to gas-food interaction. Oxygen decreased due to consumption by microorganisms, while CO_2_ decreased due to muscle absorption by seafood or meat [10,35]. A reduction of CO_2_ also occurred due to gas transfer from the packaging into the environment. They also found that higher O_2_ concentrations showed greater CO_2_ losses. However, the phenomenon of gas absorption activity requires further detailed investigation.

CO_2_ is the mainstay gas used in MAP due to its ability to maintain product quality, particularly limited microbial growth. However, CO_2_ is generally highly soluble in meat, particularly in muscle or fatty tissue, and is influenced by pH, lipid type, and lipid content. Abel et al. (2020) [120] applied NaCl in salmon to reduce CO_2_ solubility in MAP. They found that increasing NaCl concentration effectively reduced CO_2_ solubility in salmon meat, due to changes in water fraction affected by increase in electrolyte concentration, leading to salting-out phenomena and resulting in inbound and unfree water content no longer available for CO_2_ uptake.

The combination of MAP with preservation methods, such as plant extract [119], cinnamic acid [35], nisin [121], nitric oxide [122], ozonated or chlorinated water [123], and ε-polylysine [27] (Table 3), was reported to be more effective in retarding seafood deterioration and significantly delayed post-mortem metabolism rate such as ATPase activity, sarcoplasmic and myofibril degradation, lipid oxidation, biogenic amine and microbial growth.

The incorporation of hydrogen gas into MAP, called reducing atmosphere packaging (RAP), was investigated by Sezer et al. (2022) [124]. The use of RAP with 4% hydrogen significantly inhibited the formation of biogenic amines, namely heterocyclic, aromatic, and aliphatic di-amines (histamine, tyramine, putrescine, cadaverine) in rainbow trout and horse mackerel. This finding indicated that hydrogen incorporation gave advantages to biogenic amine inhibition due to the presence of antioxidant properties. Moreover, hydrogen gas is a permitted food additive with the code of E949. A patent about the application of MAP on fisheries products was registered by Jing et al. (2020) [125] for gas concentrations of 40–60% CO_2_, 0–10% O_2_, and 30–40 N_2_. They found that a gas composition of 60:5:35 of CO_2_:O_2_:N_2_ with a ratio between gas volume and puffer fish of 3:1 was optimal for packaging in terms of puffer fish quality. Packaging systems with low O_2_ and various concentrations of CO_2_, N_2_, Ar or H effectively preserved seafood quality and prevented lipid oxidation and biogenic amine production. Seafood products have different nutritional values, especially protein and lipid profiles, required specific gas concentration, gas permeability of packaging, and environmental conditions, to optimize MAP efficacy on seafood products. Modelling of MAP system for handling and storage which in turn influence shelf life of foods has been previously reviewed [126]. Future research needs to be focused on investigating, calculating, or modeling the appropriate MAP for seafood products with a specific characteristic.

### 6.2. Thermal Insulation Packaging

**Figure 7:**
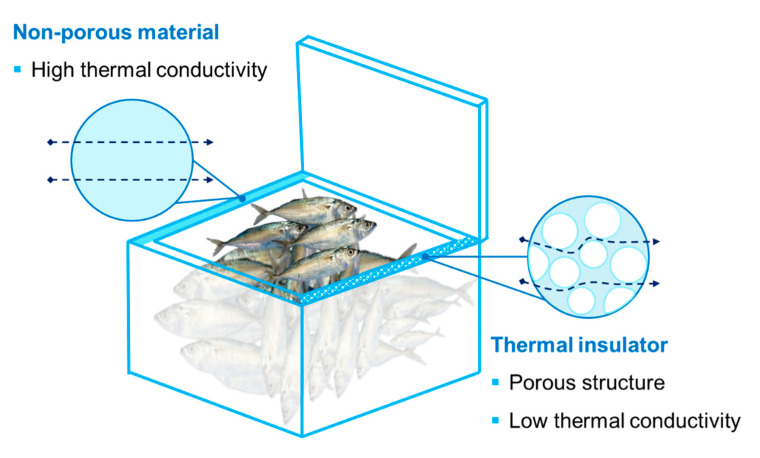
Insulator box containing numerous pores, giving low thermal conductivity to maintain the cold temperature of seafood products.

Maintaining the freshness of seafood products is important, particularly during handling and distribution. The cold chain system is the main stage for the fresh seafood product supply chain until it reaches the consumer safely. Low temperatures are mostly obtained by ice (flake ice, tube ice, block ice, crushed ice). However, packaging equipped with thermal insulation is necessary to stop the ice from melting during storage and distribution. Expanded polystyrene, polyurethane foam, and expanded polyurethane foam are common thermal insulators used for cold chain distribution [133]. They contain many pores filled with air and have very poor thermal conduction properties that inhibit the flow of heat energy (Figure 7). Sormin et al. (2016) [134] reported that Styrofoam had better cool temperature maintenance, resulting in a faster cooling rate of fish (0.13 °C/min) compared to the cool box insulator “ela sago” (0.045 °C/min). Patents for insulator boxes for fisheries’ products are listed in Table 4.

Nowadays, environmentally friendly thermal insulators have attracted research attention to replace conventional material-based products in cool chain systems. Ariany et al. (2018) [135] found that cellulose-based insulators with low thermal conductivity could be applied to fishery products to reduce the magnitude of heat flux on the fish to 61.31% of the maximum heat flux, although the system required further improvements, particularly the thickness. Thermal insulation-equipped packaging prepared from feathers had low thermal conductivity compared with expanded polystyrene [133]. The feathers were extremely lightweight and environmentally friendly, with good mechanical properties and high thermal insulation due to the hollow shaft structure. Cardboard-based thermal insulators consisting of double E-fluted corrugated board sandwiches between PE laminated and metalized E-fluted cardboard had similar thermal insulation properties to commercial expanded polystyrene; however, the complex structure of cardboard-based insulator thermal insulators requires further investigation for foldability and manufacturability performance [136]. Rigid packaging that can maintain low temperatures is required in the seafood supply chain to preserve product quality and prevent loss. Natural-based thermal insulators have started to be investigated to replace conventional polymers. Future research needs to investigate the durability of the natural-based insulator to meet the requirement for an insulator box for distribution and storage. It could be conducted by simulation with heat transfer and distribution, shock, and vibration taken into consideration.

## 7. Conclusions and Future Perspective

This paper highlighted seafood’s post-harvest deterioration and reviewed recent advances in polymeric packaging technology. Novel technology regarding polymeric active packaging, intelligent packaging, MAP, as well as rigid insulation packaging for seafood products has been developed and patented. Furthermore, the removal of the smell of seafood products related to their chemical compounds has also become a challenge in terms of packaging technology. New active compounds have been successfully used to preserve product quality, such as essential oils, nanoparticles, and plant extracts, while intelligent bioactive sensors, such as anthocyanin and metal and dye-based sensors, have been used to monitor the freshness of seafood. Biodegradable insulator packaging for seafood is at the novel development stage. From these recent results, biodegradable and sustainable polymers will be increasingly investigated for packaging applications in the future. Functionalized materials as well as improve the stability and packaging performance of biodegradable materials for seafood packaging applications will be explored by global researchers. Chemical and physical modification of polymers, lamination, coating, and blending with other sustainable materials are the technologies to improve water resistance and performance for seafood packaging. Further research is required to scale-up the polymeric-based packaging to industrial market production to meet the needs of the fishery market and promote a circular economy in the post-pandemic situation.

## Figures and Tables

**Table 1:** Previous recent research on active packaging for seafood products.

Active Agent	Polymer Materials	Seafood Product	Packaging Properties	Seafood Qualities	Source
Nanoparticles SiO, ZnO, CuO, SiO-ZnO, Si-CuO and ZnO-CuO.	Gelatin/ polyvinyl alcohol	Shrimp (*Penaeus vannamei*)	Nanoparticles exhibited antimicrobial properties against *S. aureus*, *L. monocytogenes*, *E. coli*, *P. fluorescens*, *Vibrio* and *Aeromonas*, with inhibition zone 20.1–22.1 mm and 7.2–12 mm for SiO-ZnO and CuO, respectively.	SiO-ZnO was more effective than CuO in controlling TVB-N, *Shewanella putrefaciens*, Enterobacteriaceae, and *Pseudomonas* spp. of shrimp.	[24]
ZnO, TiO_2_, or ZnO/TiO_2_	Gelatin/ polyvinyl alcohol	White shrimp	ZnO + TiO_2_ was more effective against Gram-negative bacteria than Gram-positive bacteria with the inhibition zone of 8.11 to 12.63 mm.	Active film improved shrimp shelf-life (12 days) compared to the control (6 days).	[68]
Sardinella protein isolate (SPI)	Chitosan	Shrimp	Structural and thermal properties of chitosan were improved with SPI incorporation, but it exhibited lower mechanical properties.	Shrimp packed in SPI film had psychotropic and mesophilic bacteria of 0.49 and 0.44 log CFU/g, respectively, lower than control (2.21 and 5.79 log CFU/g) on day 9.	[69]
Potato peel phenolic	Starch	Smoked sea bream fillet	Films showed a yellowish color and improvement in water tolerance, elasticity, and antioxidant activity (56–85% of ABTS inhibition).	Fillets packed in active film had a pleasant smell and flavor, an increase in golden color, and higher stiffness than fillets packed in control film.	[67]
Chitosan or lysozyme	PLA	Grass carp fillet	The film provides an antimicrobial agent to form an amine bond to inhibit *E. coli* and *S. aureus*.	Active film prolonged the fillet up to 3 days. PLA/chitosan was more effective in inhibiting bacterial growth than PLA/lysozyme.	[70]
Essential oil (Carvacrol, citral and α-terpineol)	PLA/PBAT	Pacific white shrimp	Essential oil modified barrier properties and microstructure affected by polymer-essential oil interacted via hydrogen bonding and carbonyl groups.	Shrimp deterioration was prevented by active film. Citral and carvacrol more effectively stabilized protein conformation in muscle tissues, retained drip loss and adhesion between the cephalothorax and abdomen.	[37]
Green tea ground waste	Potato starch, gelatin, carboxymethyl cellulose (CMC)	Salmon	The film had high water vapor permeability (WVP) but limited germination due to a low pH. The DPPH radical scavenging of the tray containing tea waste was 80.75%.	The active tray + film provided potential inhibition against biogenic amine accumulation, 19% lower spoilage bacteria of salmon than the control after 6 days of storage.	[71]
Carob (*Ceratonia siliqua* L.) seed macerate	Cellulose and water-based biodegradable adhesive	Atlantic salmon fillets		Salmon packaged in active film had a lower pH, drip-loss, TBARS and TVB-N up to 5 days of storage compared to the control.	[72]
*Solanum betaceum* (chilto) seed and peel extract	Pectin enriched extract	Atlantic salmon fillets	The active agent reduced mechanical properties and WVP.	Pectin containing phenolic extract showed better salmon lipid and protein oxidation protection than anthocyanin during 10 days of storage.	[73]
Maqui berry extract (MBE)	Cowpea starch	Salmon	MBE decreased rigidity, increased flexibility, UV-light blocking and antioxidant properties of cowpea starch film.	Cowpea starch film with 20% MBE retarded lipid oxidation of salmon.	[74]
Cinnamon leaf essential oil (CLE)	Bombacaceae gum	Salmon	CLE decreased tensile strength and WVP, while 1.25% CLE increased radical scavenging 1.8 times compared to the control.	Active film retarded lipid oxidation, malonaldehyde and hydroperoxide generation in salmon.	[75]
*Nostoc commune* Vauch polysaccharides (NVP)	sodium CMC	Salmon	Ratio NVP:CMC at 1:3 showed the strongest hydrogen bond and denser structure.	Salmon coated with NVP + CMC had a lower pH value, lipid and protein oxidation, preserved color and texture during 8 days of chilling storage.	[76]
*Lysozyme (LYS) and green tea extract (GTE)*	Gelatine/rice starch g-pads	Smoked salmon	Gelatin/rice starch-based g-pads mechanically improved after LYS and GTE addition, showing 1.8 and 1.7 log *Listeria innocua* reduction, respectively.	Salmon packaged with g-pads containing LYS and GTE had 1.5–1.9 log lower Listeria load than the control.	[77]
*Cinnamaldehyde* (CIN)	CMC or collagen (COL)	Tilapia		Collagen/6% CIN delayed TPC and *Vibrio parahaemolyticus* by up to 1.82 log cfu/g than control at day 14 of storage, with lower TVB-N and TBARS. Shelf-life of tilapia was 3 days longer than the control.	[78]
*Cinnamaldehyde*	Corn starch/polyvinyl alcohol	Large yellow croaker		Fish packaged in active film exhibited lower myofibril secondary and tertiary oxidation, water loss, water migration, and lipid oxidation.	[79]
*Cyclic adenosine monophosphate (cAMP)*	Red seaweed polysaccharide	Large yellow croaker	Barrier properties, surface wettability, mechanical strength, and antimicrobial activity against Gram positive and negative bacteria were promoted.	Shelf-life of large yellow croaker packaged in active film was 2 days longer than control, with lower microbial growth and TVB-N.	[80]

**Table 2:** Related research on intelligent packaging for seafood products.

Colorimetric Indicator	Packaging Material	Seafood Product	Function of Detection	Detection Response	Source
Riceberry phenolic extract	Chitosan	Shrimp	pH-sensitive, ammonia detector	Color changed from orange-red to yellow as shrimp spoilage response.	[107]
Betacyanin from paper flower	Potato starch	Caspian sprat	pH-sensitive, ammonia detector	Color changed from light pink to yellow in response to pH between 2–13 and ammonia 0.01–0.1 mg ammonia/mL water.	[108]
Betalains *Amaranthus* leaf extract	Polyvinyl alcohol and gelatin	Fish or chicken	pH-sensitive, antimicrobial activity	Color changed from red to yellow, corroborated by increased pH, TVB-N and microbial growth of meat.	[109]
Black currant anthocyanin	Konjac glucomannan and methyl cellulose	Tilapia fish	pH-sensitive	Color changed from pink-purple to yellow in response to tilapia spoilage.	[102]
Blueberry anthocyanin	Potato starch and chondroitin sulfate	shrimp	pH-sensitive	Color changed from originally pink to light grey and finally to grayish-green correlated to TVB-N, pH values and microbial profile in shrimp.	[110]
Butterfly pea (*Clitoria ternatea*) anthocyanin	Hydroxypropyl methylcellulose/microcrystalline cellulose	Mackerel (*Scomber scombrus*)	NH_3_-sensitive	Deep or light purple (fresh mackerel), violet color (mackerel suitable to eat), green to blue ocean or colonial blue (spoilage mackerel).	[111]
*Clitoria ternatea* anthocyanin	Polycaprolactone	Shrimp	pH-sensitive and ammonia detector	Color change from pale-blue to yellow-green in response to shrimp spoilage.	[112]
*Malva sylvestris* anthocyanins	PLA, polyethylene glycol (PEG), and calcium bentonite (CB)	Shrimp, fish roe, meat and chicken fillet	pH-sensitive	Color change from light red (pH 2) to green (pH 11) and more sensitive to shrimp and fish roe rather than chicken and meat correlated to TVB-N value.	[113]
Anthocyanin-rich purple potato extract	2,2 6,6-tetramethylpiperidine-1-oxyradical, oxidized bacterial cellulose and thymol	shrimp	Volatile ammonia detector	Color changed to dark purple in response to shrimp spoilage after 32 h.	[114]
Curcumin nano capsules	Soy protein isolate and cellulose nanocrystals	shrimp	pH-sensitive, ammonia detector, anti-radical scavenging	The yellow (pH 3–7) color becomes reddish-brown (pH 8–11) in response to TVB-N changes in shrimp during storage.	[103]
Curcumin	Corn starch, polyvinyl alcohol	*Pangasius bocourti* (catfish)	pH-sensitive	Color changed from yellow to orange in the range acidic (pH 3) to neutral (pH 7), and turned to red at pH 8–10 in response to TVB-N changes.	[115]
Copper nanoparticles		Salmon trout	Volatile sulfur compound	The color of white, yellow and brown as a colorimetric indicator related to fresh, semi-fresh, and spoiled salmon, respectively.	[104]
Alizarin	Gelatin and lavender essential oil	shrimp	pH-sensitive and ammonia detector, antimicrobial activity	The color changed from yellow to red-brown in response to increasing TVB-N in shrimp after 3 days of storage.	[98]
Donor-π-acceptor (D-π-A)	Cellulose	Fish	Amine detector	The color changed from red to yellow in response to putrid fish, while the emission changed to bright cyan.	[116]
Pelargonidin	Bacterial cellulose	Tilapia fillet	pH-sensitive	The color change from red to colorless in response to the TVB-N value and sensory changes of tilapia fillets.	[99]
Cyanicin-3-glucoside	Bacterial cellulose	Tilapia fillets	pH-sensitive	Color changed from red to green in pH range 3–10. During application, rose-red fresh tilapia turned to purple (acceptable) and lavender (spoilage).	[100]
Rhodamine B	AIE-stimuli-responsive polymer tetraphenylethylene (TPA) and polymethacrylic acid (PMA)	Salmon	pH-sensitive	Color change from pink (fresh) to dark blue (spoilage) was linearly correlated with TVB-N, indicating that the sensing label was feasible and non-destructive for quantitative TVB-N.	[101]

**Table 3:** Previous research on modified atmosphere packaging for seafood products.

Gas Composition	Supplementary Material	Seafood Product	Outcome	Source
Argon (Ar), nitrogen (N_2_) and carbon dioxide (CO_2_)	1% chamuang leaf extract (CLE)	Pacific white shrimp	Pulse electric field-CLE-CO_2_ treated shrimp showed the lowest pH value, carbonyl content, TVB-N, peroxide value and TBARS, and melanosis.	[119]
CO_2_:O_2_:N_2_ ratio 1) 80:5:15, 2) 60:5:35, 3) 40:5:55, 4) 20:5:75, 5) 80:15:5, 6) 60:12:25, 7) 40:15:45, 8) 20:15:65	-	Pacific white shrimp	Increased CO_2_ to 60–80% effectively reduced microbial growth, melanosis, and lipid oxidation. O_2_ decreased with increasing CO_2_ during storage due to microbial growth. O_2_ and CO_2_ decreased due to consumption by microbials and dissolved in shrimp meat, respectively.	[10]
75% CO_2_ and 25% N_2_	Oregano 0.1%, nisin 0.2%, oregano 0.1% + nisin 0.2%	Grass carp (*Ctenopharyngodon idellus*)	Nisin and oregano EO showed synergistic antimicrobial effects, which extended the shelf-life of fish fillets up to 28 days, with lower microbial growth and tyramine levels and increased pH values.	[121]
60% CO_2_/40% N_2_	Skin vacuum packaging: Skintite HB 125 alu/pet (PE/EVOH combination)	Atlantic salmon portions	MAP showed comparable results with skin packaging in terms of drip loss, water holding capacity, texture, and microbial count. Salmon packaged in MAP and skin packaging extended microbial shelf-life by 1.5 times compared to the control.	[127]
60% CO_2_/5% O_2_/ 35% N_2_	ε-polylysine (0.1%, 0.2%, 0.3%)	Pufferfish (*Takifugu obscurus*)	ε-polylysine, chitosan and sodium alginate coatings and MAP delayed myofibril oxidation, preserved Ca^2+^-ATPase activity, α-helix and β-sheet contents, and stabilized tertiary structure during cold storage.	[27]
MAP1: 50% CO_2_/50% N_2_ MAP_2_: 60% CO_2_/40% N_2_ RAP1: 50% CO_2_/46% N_2_/4% H_2A_ RAP2: 60% CO_2_/36% N_2_/4% H_2_	-	Rainbow trout and horse mackerel	Reduction rates of biogenic amine in fish packaged following the reducing atmosphere packaging (RAP) technique were 2 times higher than in MAP, indicating the efficiency of hydrogen incorporation to prevent biogenic amine formation.	[124]
60% CO_2_/40% N_2_	Non-carbonated 10% NaCl-brine Carbonated water Carbonated 10% NaCl-brine	Salmon (*Salmo salar* L.)	Increasing NaCl concentration reduced CO_2_ solubility in salmon according to Henry’s constant and CO_2_ absorption within the salmon.	[120]
60% CO_2_/30% Ar/10% O_2_ (cold plasma/CP)	Ethanolic coconut husk extract (ECHE) Liposomal encapsulated ECHE (LE-ECHE)	Asian sea bass (*Lates calcalifer*)	Cold plasma-treated fish enriched with ECHE and LE-ECHE were 1 log cfu/g lower than control. ECHE and LE-ECHE treated fish exhibited lower protein and lipid oxidation compared to CP only.	[128]
40% CO_2_/60% N_2_	Superchilling condition	Atlantic cod (*Gadus morhua* L.)	A 1.7 °C superchilling temperature with MAP 35% CO_2_ condition effectively extended the shelf-life of Atlantic cod by up to 32 days.	[129]
80% Ar/20% O_2_	Chitooligosaccharide (COS) from squid pen	Yellowfin tuna	COS 400 ppm combined with MAP preserved the redness of tuna with lowest metmyoglobin content during storage, oxygen-based MAP showed highest lipid oxidation.	[130]
67% CO_2_/ 33% O_2_ or N_2_	-	Saithe fillets	Combination CO_2_/O_2_ with inoculated *P. phosphoreum* and *Shewanella* sp. exhibited the highest H-value, hypoxanthine, and TMA level of saithe fillets.	[131]
60% CO_2_/30%Ar/10% O_2_	Betel (*Piper betle* L.) leaf ethanolic extract (BLEE) Liposome loaded BLEE (L/BLEE)	Tilapia slices	L/BLEE at 400 ppm/MAP/non-thermal plasma treatment effectively extended the shelf-life of tilapia slices up to 12 days, related to deformation and perforation of the cell wall of bacteria observed via SEM.	[132]

**Table 4:** Patents related to thermal insulators and box packaging for fishery products.

Patent Title	Invention Details	Application	Patent Source
Containing bag for fresh fish	A bag contained waterproof material at the outer most portion, equipped with outer heat insulating material made from glass wool and nonwoven fabric. The cooling system, prepared from nitrogen gas sealed in the hollow layer reached cooling temperature of −1 °C to −5 °C. The mouth of the bag was equipped with fastener material (Velcro fastener or waterproof fastener).	Transportation and storage bag for high-economical commodity, particularly tuna and camellia in cold insulation state.	JP1995243741 [45]
Portable cool box	The component of the cooling box was hollow with a rectangular parallelepiped case body, a lid, cold insulator, and support mechanism. The insulator material contained highly water-absorbing polymer sodium polyacrylate sealed in a hollow plastic case to maintain high water absorbency by forming a gel structure.	Portable cool box for retaining the freshness of fish or other objects which can be easily used in fishing or camping.	JP2013085550 [46]
Intermediate dish of foamed fish box	The overlapped box containing two outer boxes and an upper layer made from cold resistant olefin sheet-based raw material. The depth of the main container was adjusted by calculating the amount of ice required. An edge over the upper layer created an overlapped insulator box.	Container for fresh fish at processing site or for transportation.	JP2018135150 [47]
Cooling packing box	A Styrofoam-based insulator box equipped with an intermediate lid attached to the upper end of the main body. The intermediate lid had storage space for frozen food products in the middle, with holes to allow cold air to pass through the storage space in the intermediate lid.	Non-direct contact cooling system using cold air for fresh products.	KR1020180112388 [48]
Corrugated carton suitable for transportation and packaging of fish tank	A corrugated box for transporting fish tanks equipped with an inner and outer groove on the foam base. The inner groove is equipped with a rubber sleeve and the fish tank is placed on the outside of the rubber sleeve on the outer groove. Between the fish tank and rubber sleeve, rubber supporting feet are attached with a soft rubber cushion, EPE pads protect the fish tank and are reusable.	Corrugated paper box for fish tank transportation and packaging.	CN209427201 [49]
Circular fish tank buffering packaging box formed by one piece of paper	A fish box formed by one piece of paper packaging as the main body, with linings and a handle, comprising a bottom plate, four end plates, a top plate, four lining plates and a bottom plate. The handle comprises two triangular top plates. Prevents fish transportation damage with reduced amounts of foam, reduced production cost, and material saving.	Paper box for fish transportation to minimize damage.	CN209720191 [137]
Packaging box for refrigerating fresh fish meat	A box with an inside structure, including an inflatable bag with an inflatable interface on the top, foam plastic board, absorbent paper made from wood pulp layer and a non-woven layer on both sides, and a wave-shaped convex strip in the inner wall. A sealing gasket is attached between the box cover and box body, with a support pad placed in the bottom of the box.	Packaging box for refrigerating fresh fish meat.	CN215246117 [138]

## Data Availability

The data presented in this study are available on request from the corresponding author.
